# Subunit Vaccine Approaches for African Swine Fever Virus

**DOI:** 10.3390/vaccines7020056

**Published:** 2019-06-25

**Authors:** Natasha N. Gaudreault, Juergen A. Richt

**Affiliations:** Department of Diagnostic Medicine & Pathobiology, College of Veterinary Medicine, Kansas State University, K224 Mosier Hall, 1800 Denison Ave, Manhattan, KS 66506, USA

**Keywords:** African swine fever virus, ASFV, vaccines, subunit vaccines, antigens, immunogens, protective immunity, disease enhancement, antibody dependent enhancement

## Abstract

African swine fever virus (ASFV) is the cause of a highly fatal disease in swine, for which there is no available vaccine. The disease is highly contagious and poses a serious threat to the swine industry worldwide. Since its introduction to the Caucasus region in 2007, a highly virulent, genotype II strain of ASFV has continued to circulate and spread into Eastern Europe and Russia, and most recently into Western Europe, China, and various countries of Southeast Asia. This review summarizes various ASFV vaccine strategies that have been investigated, with focus on antigen-, DNA-, and virus vector-based vaccines. Known ASFV antigens and the determinants of protection against ASFV versus immunopathological enhancement of infection and disease are also discussed.

## 1. Introduction

African swine fever virus (ASFV) causes a devastating and economically significant disease of both domestic and wild swine (Sus scrofa). There is no available vaccine for ASFV, and current control methods involve quarantine and culling of animals in affected areas and regions. ASFV is found in Africa, where it is maintained in a sylvatic cycle between soft ticks, warthogs, and bushpigs which do not develop disease with ASFV infection [1]. On the other hand, ASFV can cause high morbidity and mortality in domestic pigs and wild boar [2,3]. 

ASFV is endemic in Africa, where it was first described in the early 1900s [4,5]. In 1957, ASFV emerged outside of Africa in Portugal and from 1960 to the 1980s subsequently spread across Western Europe. In the 1970s to early 1980s, ASFV emerged in the Caribbean islands and Brazil. By the mid-1990s, ASFV had been eradicated in the Americas and Europe, with the exception of Sardinia, which has remained endemic since 1982 [4,5]. In 2007, ASFV was introduced to the Caucasus region and quickly spread into the Russian Federation and Eastern Europe, where it has continued to circulate [4,5,6,7,8]. More recently, ASFV has been found as far west as Belgium in wild boar [9]. Currently, ASFV is circulating in domestic swine of China and has spread to surrounding countries, including Mongolia, Vietnam, Cambodia, Laos and North Korea (DPRK) [10,11]. There are currently 24 genotypes of ASFV based on the major capsid protein p72, and 8 serotypes based on the viral hemagglutinin CD2-like protein (CD2v) and C-type lectin [12,13,14,15]. The virus circulating in Europe, Russia, and China has been identified as a highly virulent, genotype II strain [16,17,18,19]. ASFV is highly contagious and stable in the environment and can be readily transmitted through infected pork products and contaminated fomites [20,21,22,23,24]. Thus, ASFV poses a significant threat to the swine industry worldwide, and the need for an ASFV vaccine is of high priority. 

The pursuit for an effective vaccine against ASFV has been largely unsuccessful. This is due to the complexity of the virus and our limited understanding of ASFV virulence factors and the correlates of protection. ASFV, the sole member of the family *Asfarviridae*, has a large double-stranded DNA genome ranging in size from 170 to 190+ kb depending on the strain, and encodes more than 150 proteins, many of which remain uncharacterized [25,26,27,28]. Up to 68 structural proteins have been identified from the virion alone [29]. Identified ASFV antigens and immunogens, and ASFV targets investigated for vaccine development are discussed later in this review.

## 2. Inactivated and Live Attenuated ASFV Vaccines 

Conventional vaccine approaches such as inactivated virus have proven to be ineffective [30,31]. More success has been seen with naturally attenuated isolates [32,33] or modified live viruses [33,34,35,36,37,38,39]. However, protection is generally only against homologous strains of the same genotype, and not heterologous virus challenge. Furthermore, live attenuated strains often have associated adverse side-effects, such as skin lesions and joint swelling, which have hindered their development as vaccines [32,40,41,42,43]. Live virus vaccines can also cause chronic or persistent infections and have the potential to revert to virulence. Another issue with live attenuated vaccines is the lack of a stable cell line for production, since ASFV preferentially replicates in primary monocyte/macrophage cells. For these reasons, subunit and vectored ASFV vaccines have been explored as an alternative viable option. 

## 3. Subunit, DNA, and Virus-Vectored ASFV Vaccines

Several subunit, DNA, and virus vector vaccine strategies have been investigated with limited success and sometimes inconsistent results. This inconsistency could be attributed to a variety of factors, including the type of vaccine, vaccination strategy, the antigens used, and the immune response induced, as well as the challenge model used, including factors like animal genetics, virus strain, and vaccine and challenge dose. Antigen- and DNA-based vaccines provide a targeted approach with fewer side-effects and increased safety compared to live or inactivated virus vaccines. A number of immunogenic ASFV proteins have been identified and investigated for a role in protection against ASF, which are summarized and discussed later in this review. ASFV structural proteins p30, p54, p72, pp62, and CD2v encoded by genes CP204L, E183L, B646L, CP530R, and EP402R, respectively, have been the main targets of subunit and DNA vaccine strategies, including vaccination with either individual ASFV antigen targets or as multitarget cocktails. The following subsections summarize the subunit, DNA, and virus-vectored ASFV vaccines evaluated thus far.

### 3.1. Antigen-Based Vaccines

Earlier ASFV vaccine studies focused on antigen-based approaches, aimed at inducing neutralizing serological responses (Table 1). The first recombinant ASFV protein to demonstrate protection against ASFV challenge was the baculovirus-expressed ASFV hemagglutinin (HA) protein, CD2v [44]. Pigs vaccinated 3 times with recombinant CD2v proteins, then challenged with the virulent ASFV genotype I E75 strain, produced CD2v-specific antibodies, with one pig exhibiting virus-neutralizing activity. All three immunized pigs were protected from lethal challenge, although two animals did become viremic. This study, along with previous results, indicated that CD2v was not a strong immunogenic antigen, and high doses of the protein would likely be required to induce good protection [45].

Many of the other subunit vaccine approaches since have focused on ASFV p54 and p30, two structural proteins involved in virus attachment and internalization, respectively, both of which are capable of inducing virus neutralizing antibodies [46]. Immunization of pigs with p54 or p30 antigen alone was not sufficient to protect pigs against virulent ASF challenge. However, pigs immunized with both, p54 and p30 together had a delay in onset of clinical symptoms, reduced viremia, and 3 out of 6 pigs were protected from virulent challenge with the E75 strain [46]. Similarly, immunization with a baculovirus-expressed p54/p30 fusion protein also reduced viremia and protected all pigs against virulent challenge with E75 [47]. 

On the other hand, pigs immunized with a cocktail containing baculovirus-expressed proteins p30 + p54 + p72 + p22 were not protected from homologous challenge with the virulent ASFV Pr4 genotype I strain [48]. The results of that study indicated that neutralizing antibodies induced by these proteins are not sufficient for protection. This was supported by the earlier study with recombinant CD2v, in which protection in the absence of neutralizing antibodies was observed [44]. In line with these results, which indicated that factors other than neutralizing antibodies play a role in ASFV protection [44,48], a study by Oura and colleagues demonstrated the importance of the cell-mediated, cytotoxic T lymphocyte (CTL) immune response for ASF protection [50]. In that study, pigs immunized with an attenuated ASFV strain and depleted of CD8+ T cells were not protected from homologous virulent challenge whereas non-depleted pigs were protected. Together, these results indicate that in addition to antibodies, a vaccine capable of stimulating a T cell-mediated response is likely required for conferring protection against ASFV.

### 3.2. DNA Vaccines

In contrast to antigen-based subunit vaccines, DNA vaccines are capable of inducing cell-mediated CTL immune responses, shown to play an important role in protection against ASFV [50]. As with ASFV subunit vaccine formulations, p54 and p30 have also been the target antigens for DNA vaccine approaches (Table 2). 

Vaccination with a plasmid DNA encoding the p54/p30 fusion protein produced neither neutralizing nor T cell responses and was not protective against challenge [51]. Pigs vaccinated with a DNA vaccine construct encoding a fusion of the swine leukocyte antigen SLA-II with p54/p30 developed broad immunological responses, including both specific antibodies and T cells, but were also not protected from challenge [52]. In an attempt to induce protection previously observed with the subunit vaccine of a similar formulation [47], the extracellular, soluble domain of the ASFV hemagglutinin protein, CD2v (designated as sHA), was fused to the p54/p30 chimera [51]. Immunization with the sHA/p54/p30 construct did induce antigen-specific B- and T cell responses, but no protection was observed. However, the addition of ubiquitin to the sHA/p54/p30 fusion construct to target class I antigen presentation in vivo did confer protection against challenge in a small percentage of immunized pigs. Survival correlated with presence of T cells in the absence of detectable neutralizing antibodies, further supporting the importance of CTLs in protection against ASFV.

A DNA expression library established to identify T cell targets involved in ASFV protective immunity further supported the importance of CTL response in protection [53]. The library consisted of 80 ASFV open reading frames based on the genotype I Ba71v strain, fused with ubiquitin, representing approximately half of ASFV encoded proteins. Immunization of pigs with the DNA library conferred 60% protection against lethal challenge with the E75 strain. No specific anti-ASFV antibodies were detected following vaccination but were detectable after challenge along with ASFV-specific T cells.

### 3.3. Virus Vectored Vaccines

Another strategy to elicit both humoral and cell-mediated immune responses is the use of viral vectors. Safety is ensured by removing or replacing virulence genes of respective viruses with immunogens or making the virus vector replication incompetent. Moreover, viral vectors are inherently compatible for differentiating infected from vaccinated animals (DIVA), i.e., the virus vector encoded immunogens can serve as vaccine markers. To date, several vector-based approaches have been evaluated in pigs, yet few have been tested against virulent ASFV challenge (Table 3). A BacMam vector used for delivery of the sHA/p54/p30 fusion construct to pigs provided protection against sub-lethal challenge in 4 out of 6 pigs [54]. BacMam is a baculovirus-based vector with the ability to transduce mammalian cells to express the target genes. In the above study, no specific antibody response was detected, but protection correlated with a strong virus-specific T cell response.

Alphavirus replicon particles (RPs) expressing p30, p54, and p72 have also been used to immunize pigs [55]. Vaccinated pigs developed strong antibodies against p30, and positive virus neutralization with sera from pigs immunized with p30 suggested a low level of neutralizing activity. Alphavirus-p54 vaccinated pigs developed low levels of anti-p54 antibodies, and no antigen-specific antibodies were detected from sera of p72 immunized pigs. However, the addition of the sHA domain of CD2v to p72 did result in detectable levels of antibodies against p72. Expression levels of each of the antigens in vitro correlated with the immune responses generated. Unfortunately, this study did not include data on cell-mediated immune responses, and protection against virulent challenge was not tested.

Immunogenicity studies with antigen cocktails delivered by virus vectors have also been evaluated in swine. Cocktails of adenovirus delivered ASFV antigens p30 + p54 + p72 + pp62, and ASFV genes A151R + B119L + B602L + EP402RΔPRR + B438L + K205R + A104R induced both strong antigen-specific humoral and cellular immune responses [56,57]. Another ASF vaccine cocktail consisted of modified Vaccinia virus Ankara-vectored ASFV antigens p72, CD2v, and C-type lectin [49]. Although no antigen-specific antibodies were induced, T cell responses for each of the antigens were detected in immunized pigs. However, none of these immunization studies were tested against virulent virus challenge.

A mouse model was used to evaluate a recombinant Newcastle disease virus expressing p72 and was shown to be safe and immunogenic [58]. Yet it is difficult to predict how these results translate to swine, as observed previously with a p54/p30 DNA vaccine, which was found to be immunogenic in the mouse model but not in pigs [51,52]. This highlights the importance of evaluation of vaccine prototypes using the target animal species, the pig.

### 3.4. Combination and Heterologous Prime-Boost Vaccination Approaches

Combination vaccines and heterologous prime-boost strategies which incorporate the use of two different vaccine platforms to induce better humoral and cellular immune responses have also been investigated and are summarized in Table 4. One such approach is a combined DNA–protein vaccine. Immunogenicity of various combinations of antigens and plasmid DNAs in swine was determined [59], and the antigens which induced neutralizing antibodies and T cell responses were subsequently tested as a DNA–protein vaccine cocktail in challenge experiments [60]. The vaccine cocktail contained 7 different ASFV targets—p15, p35, p54, p72, CD2v, p30, and p17—and was delivered 3 times prior to challenge. Vaccinated pigs produced antibodies against ASFV antigens p15, p35, and p54, but no neutralizing activity was observed. Only few antigen-specific T cells were detected after vaccination, and pigs were not protected following lethal challenge with the Armenia 2007 strain [60].

Other approaches have incorporated virus vector and DNA or protein-based vaccine platforms. A heterologous prime–boost approach, consisting of priming pigs with 47 plasmid DNA constructs and boosting with 47 recombinant vaccinia viruses, was employed to identify potential protective immunogens [61]. A total of 47 antigens were represented and tested for the ability to induce humoral and cellular immune responses. T cell responses and specific antibodies were detected for many of the antigens, but no neutralizing activity was detected, even in the presence of anti-p30, -p54 and -p72 antibodies. Importantly, the number of antigens included in the cocktails did not appear to affect responses to individual antigens. Vaccinated pigs were tested against virulent challenge with the genotype II Georgia 2007/1 strain. Although none of the vaccinated pigs were protected, virus levels were reduced in blood and certain tissues [61]. 

A modified Vaccinia Ankara virus vector expressing individual p72, CD2v, and C-type lectin ASFV antigens, followed by a boost with the corresponding mammalian cell-expressed proteins, also yielded T cell responses against each of the antigens, particularly for p72 [49]. However, protection against virus challenge was not tested. Finally, a heterologous prime–boost approach incorporating the alphavirus delivered ASFV p30 and an attenuated ASFV strain induced a strong anti-p30 antibody response as well as had the capacity to at least partially neutralize virus infection in vitro; however, T cell responses and protection against virus challenge were not evaluated in that study [55]. Evaluating these vaccination studies against virulent ASFV challenge in swine will be critical to determine the true protective potential of each of these strategies.

## 4. Immune Determinants of Protection against ASFV

The correlates of protection against ASFV are still not completely understood. Results for the role of ASFV neutralizing antibodies in protection are somewhat conflicting [62]. A strong neutralizing antibody response has been associated with protection [46,47] but does not appear to define it, since protection was also achieved the absence of neutralizing antibodies [44,51,53,54]. Furthermore, the presence of neutralizing antibodies in another study was shown not to be sufficient for protection [48]. Exacerbation of ASFV infection and disease in immunized pigs associated with high levels of non-neutralizing antibodies has also been documented and is discussed in the next section. 

A study by Oura and colleagues demonstrated that CD8+ T cells play an important role in ASFV protection, and a clear correlation exists between protection and the presence of ASFV-specific T cells [50,51,53,54]. Natural killer (NK) cells also appear to play a role in protection. High levels of NK cell activity in pigs immunized with the naturally attenuated, nonhemadsorbing NH/P68 strain correlated with protection against virulent challenge [40]. Thus, both neutralizing antibodies and strong cell-mediated immune responses may be important for protection against ASFV.

### 4.1. ASFV Antigen Targets

Identification of ASFV targets that play a role in protection is important for the development of an effective vaccine against ASFV. Several immunogenic ASFV targets have been identified. Still, it is not completely clear which ones play a significant role in protection against ASFV. Table 5 shows ASFV antigens identified by reactivity with sera from ASFV infected swine. Of 14 viral proteins initially found to be immune reactive against infected sera from domestic swine and bush pigs [63], 12 were further tested in a longitudinal serological study with pigs infected with the attenuated NH/P68 strain [41]. Those studies showed overall poor antibody responses to the following ASFV recombinant proteins: K196R/thymidine kinase, K78R/p10, C44L, intermediate antibody responses to B646L/p72, CP204L/p30, CP312R, NP419L/DNA ligase, and F334L/ribonucleotide reductase; and strong responses to E183L/p54, K205R, A104R/viral histone, and B602L/p72 chaperone, the latter of which were further evaluated and antigenicity confirmed with sera from swine infected with virulent virus [62]. 

Of these viral proteins recognized by infected pig sera, p30, p54, p72, A104R, B602L, NP419L, and K205R have been further investigated in immunogenicity and vaccine studies, in addition to a number of other ASFV targets. Table 5 summarizes ASFV targets that have been used in various vaccine formulations and which have been shown to induce either antibody or cell-mediated responses, or both. The main ASFV antigens known to induce neutralizing antibodies are p72, p54, and p30 [46,48,65,66,67] and are also the most extensively investigated ASFV antigens in terms of immunogenicity studies and for development of ASFV vaccines and diagnostics. Other antigens/immunogens confirmed by multiple studies include CP530R/pp62 and its derivate p15, EP402R/CD2v, B602L/p72 chaperone, and EP153R/C-type lectin (see Table 6). 

An immunogenicity screen of 47 different ASFV antigens found that previously identified CP204L/p30, E183L/p54, B602L/p72 chaperone, CP530R/pp62, and newly identified EP364R, F317L, MGF505-4R, MGF360-11L, CP2475L/pp220, E119L/virion protein, F1055L/helicase, G1211R/DNA polymerase, and NP1450L/RNA polymerase 1 to consistently induce high cellular immune responses [61]. Antigens CP204L/p30, D117L/p17, EP153R/C-type lectin, and L10L/KP117R-related protein consistently induced high levels of antigen-specific antibodies; however, no neutralizing activity was detected despite the presence of specific antibodies to known neutralizing antigens p30, p54, and p72.

### 4.2. ASFV Immune-Mediated Enhancement of Disease

Several vaccine-challenge studies suggest immune-mediated enhancement of ASFV infection and disease. Which factors are responsible is not clearly understood, but high levels of antibodies appear to play a role, and several ASFV immunogens have been associated with enhanced infection and/or pathology (Table 7). Elevated antibody levels in pigs developing chronic ASFV have been demonstrated previously [68], and systemic immune overstimulation appears to be associated with chronic or persistent ASFV infections [69]. In a study by Leitao et al. (2001), pigs immunized with the attenuated NH/P68 strain were divided into 2 groups based on clinical and immunological criteria: asymptomatic animals versus those that developed chronic clinical disease [40]. Clear differences were observed between ASFV-specific antibody titers and the level of NK cell activity of the two clinically defined groups. Asymptomatic pigs had high NK cell activity but relatively low anti-ASFV antibodies. By contrast, animals that developed chronic type lesions exhibited late fever and viremia, had high levels of ASFV-specific antibodies, and relatively normal NK cell activity levels. The elevated antibody levels observed involved IgG1, IgG2, IgM, and IgA subclasses of immunoglobulins. In another study which screened serological responses of NH/P68 infected pigs against 12 ASFV recombinant antigens, higher antibody titers against ASFV targets NP419L, CP312R, K196R, K205R, and especially p72 appeared to correlate with the occurrence of lesions observed in chronically infected animals [41]. Interestingly, higher antibody levels, including total IgG as well as IgG1, IgG2, and IgM responses against A104R tended to be associated with asymptomatic pigs.

Pigs vaccinated with a DNA vaccine construct encoding a fusion of the swine leukocyte antigen SLA-II and ASFV p54 and p30 developed broad immunological responses, including both antigen-specific antibodies and T cells [52]. However, following challenge, vaccinated pigs were not protected and had statistically higher viremia compared to the control animals, especially at 3 days post-infection. Furthermore, immune sera from the vaccinated animals did not neutralize but appeared to enhance infection of macrophages in vitro.

A DNA chimera of the extracellular domain of ASFV CD2v (sHA), p54, and p30 induced specific, non-neutralizing antibodies but did not confer protection [51]. However, the addition of ubiquitin (Ub) to the fusion construct modified the induction of immune responses in vaccinated pigs and conferred partial protection, which correlated with activated T cell responses [51]. Hyperimmunization also appeared to be associated with reduced protection. Although not statistically significant, four doses of the DNA Ub/sHA/p54/p30 construct resulted in fewer pigs protected from challenge, as compared to two immunizations of the same vaccine (Table 2), which was observed in two independent experiments [51]. The hypothesis was that the extra vaccination may have induced low levels of immunopathological antibodies that may have in turn reduced the number of pigs protected from challenge [51].

Statistically higher clinical scores were observed in immunized pigs with ASFV antigen pools by DNA prime and boost with recombinant vaccinia virus compared to control pigs [61]. Immunization induced both antibody and T cell responses and also reduced viral loads in the blood and some tissues, but pigs were not protected from challenge. No virus neutralizing activity was detected.

In an ASFV vaccine study evaluating adjuvants combined with inactivated virus, high antibody titers that lacked neutralizing activity correlated with an accelerated disease course [31]. Vaccinees were not protected following homologous ASFV challenge, and in fact, enhanced infection and disease was observed. Similarly, pigs immunized with a cocktail of ASFV DNA and ASFV proteins also had an accelerated disease course compared to nonvaccinated controls [60]. Vaccinated pigs had earlier onset of clinical symptoms, viremia, and death following lethal challenge. Pathological scores also tended to be higher in vaccinated versus control pigs. Immunological analyses showed vaccination induced minimal T cell responses but detectable levels of antigen-specific antibodies, which were unable to neutralize virus and instead enhanced ASFV infection in vitro [60].

Taken together, the results of these studies suggest that an overproduction of antibodies is likely detrimental and exacerbates disease progression, especially in the absence of a strong cell-mediated immune response. However, it is still not clear whether neutralizing and/or non-neutralizing antibodies are responsible for enhancement of infection and disease. One possible explanation is antibody-dependent enhancement (ADE) of infection, mediated via IgG antibody-antigen complexes and Fcγ-receptor signaling, which is known to occur with microorganisms that replicate in macrophages, like ASFV, porcine reproductive and respiratory virus, dengue virus, and a number of other viral pathogens [70,71,72,73]. Studies to investigate ASFV immune enhancement and the mechanism involved in ASFV pathogenesis are warranted and will be beneficial for the development of safer and more effective ASFV vaccines.

## 5. Conclusions

Various vaccine strategies for ASF have been investigated with a wide variety of ASFV-specific targets evaluated. Yet, many of these strategies have not been tested against virus challenge. Which ASFV targets play a significant role in virulence and immunopathology or protection is still largely unknown. Expanding our understanding of the ASFV proteome and the functions of individual proteins will be important for rationally designing targeted vaccine approaches. Furthermore, finding the right balance between both antibody- and cell-mediated ASFV immune responses is clearly important. Immune overstimulation appears to be the key factor affecting the disease course of ASF, and high levels of antibodies appear to have a particularly detrimental effect on clinical outcome and protection. Shifting the focus to less immunogenic ASFV antigens and the identification of novel neutralizing ASFV antigens or epitopes may also prove to be beneficial. For example, p30 is capable of inducing a strong antibody response; however, only a portion of these antibodies appear to be virus neutralizing. Determining which type of antibodies are detrimental and perhaps the identification of neutralizing and non-neutralizing epitopes within individual antigens could be beneficial for designing a more targeted immune response to improve protection. Nonetheless, induction of strong cell-mediated immunity, such as NK and T cell responses, appears to remain a critical component for designing a safe and efficacious ASFV vaccine.

## Figures and Tables

**Table 1:** Antigen-based African swine fever virus (ASFV) vaccines evaluated in the swine model.

Vaccine Type	ASFV Target Protein (Strain)	Number of Immunizations; Dose, Adjuvant	Specific/Neutralizing Antibodies	T Cell Response	Challenge Strain; Dose	Clinical Outcome	Ref.
Baculovirus-expressed proteins	CD2v (E75CV)	3×; 0.5–1 × 10^7^ HAU + Freund’s adjuvant	Yes; No	NA	E75; 4 × 10^2^	100% protection, *n* = 3/3	[44]
Baculovirus-expressed proteins	p30, p54, p54 + p30 (E75)	3×; 100 μg + Freund’s adjuvant	Yes; Yes	NA	E75; 5 × 10^2^	50% protection, *n* = 3/6	[46]
Baculovirus-expressed proteins	p54/p30 chimera (E75)	5×; 100 μg + Freund’s adjuvant	Yes; Yes	NA	E75; 5 × 10^2^	100% protection, *n* = 2/2	[47]
Baculovirus-expressed proteins	p54 + p30 + p72 + p22 (Pr4)	4×; 200 μg + Freund’s adjuvant	Yes; Yes	NA	Pr4; 10^4^	Slight delay of clinical disease and viremia; No protection, (*n* = 0/6)	[48]
HEK cell-expressed proteins	p72, p54, p12 (Georgia 2007/1)	2×; 200 μg/antigen + TS6 adjuvant	Yes; NA	Some	NA	NA	[49]

p30 also referred to as p32; CD2v also referred to as HA = hemagglutinin; NA = not available.

**Table 2:** DNA-based ASFV vaccines evaluated in the swine model.

Vaccine Type	ASFV Target Protein (Strain)	Number of Immunizations; Dose	Specific/Neutralizing Antibodies	T Cell Response	Challenge Strain; Dose	Clinical Outcome	Ref.
DNA (pCMV)	p54/p30 fusion (E75)	3×; 600 μg	No; NA	No	E75; 10^4^	No protection, (*n* = 0/4; *n* = 0/4)	[51,52]
DNA (pCMV)	SLA-II/p54/p30 fusion (E75)	3×; 600 μg	Yes; No	Yes	E75; 10^4^	No protection, (*n* = 0/4)	[52]
DNA (pCMV)	sHA/p54/p30 fusion (E75)	3× and 4×; 600 μg	Yes; No	Yes	E75; 10^4^	No protection, (*n* = 0/6)	[51]
DNA (pCMV)	Ub/sHA/p54/p30 fusion (E75)	2× and 4×; 600 μg	Not detectable	Yes	E75; 10^4^	Partial protection, (2 immunizations, *n* = 2/6; 4 immunizations, *n* = 1/6)	[51]
DNA expression library	80 ORFs fragments fused with Ub (Ba71V)	2×; 600 μg	Yes-after challenge; NA	Yes-after challenge	E75; 10^4^	60% protection, (*n* = 6/10)	[53]

p30 also referred to as p32; CD2v also referred to as HA = hemagglutinin; sHA = extracellular/soluble domain; Ub = cellular ubiquitin; SLAII = swine leukocyte antigen class II DR molecule; pCMV = plasmid under cytomegalovirus promotor; ORFs = open reading frames; NA = not available.

**Table 3:** Virus vector-based ASFV vaccines evaluated in the swine model.

Vaccine Type	ASFV Target Protein (Strain)	Number of Immunizations; Dose, Adjuvant	Specific/Neutralizing Antibodies	T Cell Response	Challenge Strain; Dose	Clinical Outcome	Ref.
BacMam	sHA/p54/p30 fusion (E75)	3×; 10^7^ PFU	No (only after challenge); No	Yes	E75; 2x sublethal challenge 10^2^	Partial protection, (*n* = 4/6)	[54]
Adenovirus	p30+p54+pp62+p72 (Georgia 2007/1)	2×; 10^10^ or 10^11^ per Ad5-antigen + adjuvants	Yes; NA	Yes	NA	NA	[56]
Adenovirus	A151R+B119L+B602L+EP402RΔPRR+B438L+K205R+A104R (Georgia 2007/1)	2×; 10^11^ per Ad5-antigen + adjuvant	Yes; NA	Yes	NA	NA	[57]
Vaccinia virus Ankara	p72, C-type Lectin, CD2v (Georgia 2007/1)	2×; rVACV-ASFV 10^7^ TCID_50_	No; NA	Yes	NA	NA	[49]
Alphavirus RPs	p30, p54, p72, sHA/72 (Ba71V)	3×: 2-4.5 × 10^7^ RPs	Yes; NA	NA	NA	NA	[55]

p30 also referred to as p32; CD2v also referred to as HA = hemagglutinin; sHA = extracellular/soluble domain; rVACV = recombinant vaccinia virus; RPs = replicon particles; NA = not available.

**Table 4:** Combination and heterologous prime-boost ASFV vaccine strategies.

Vaccine Type	ASFV Target Protein (Strain)	Number of Immunizations; Dose, Adjuvant	Specific/Neutralizing Antibodies	T Cell Response	Challenge Strain; Dose	Clinical Outcome	Ref.
Combination							
DNA–Protein	Combinations of DNA and protein: p15, p30, p35, p54, p72, CD2v, CP312R, g5R (Georgia 2007/1; Ba71V)	3×; 100 μg per DNA, 100 μg protein + ISA25 adjuvant	Yes; Yes	Some	NA	NA	[59]
DNA–Protein	Proteins: p15, p35, p54, p17; DNA: CD2v, p72, p54, p30, p17 (Georgia 2007/1; Ba71V)	3×; 100 μg per DNA, 100 μg protein + ISA25 adjuvant	Yes; No	Some	Armenia 2007; 360 HAU	No protection; disease enhancement	[60]
Heterologous Prime-Boost							
DNA prime + vaccinia virus boost	47 antigens (Georgia 2007/1)	Prime 2×: 10 μg pCMV-DNA + CpG oligo adjuvant; Boost 2×: 10^8^ PFU rVACV-ASFV	Yes; No	Yes	Georgia 2007/1; 10^4^	No protection; reduced viral load, higher clinical scores	[61]
Vaccinia virus prime + protein boost	p72, C-type Lectin, CD2v (Georgia 2007/1)	Prime: rVACV-ASFV 10^7^ TCID_50_; Boost: 200 μg/antigen + TS6 adjuvant	NA	Yes	NA	NA	[49]
Alphavirus RP prime + live attenuated ASFV boost	p30 (Ba71V) + OURT88/3	Prime 2×: 2-4.5 × 10^7^ RPs; Boost: 10^4^ TCID_50_ OURT88/3	Yes; Yes	NA	NA	NA	[55]

p30 also referred to as p32; CD2v also referred to as HA = hemagglutinin; sHA = extracellular/soluble domain; rVACV = recombinant vaccinia virus; RPs = replicon particles; NA = not available.

**Table 5:** Antigens recognized by ASFV infected pig sera.

ASFV Gene	Product	ASFV Strains	IgG	IgM	Ref.
Structural proteins					
A104R	Viral histone-like	Malta, Malawi, OURT88; NH/P68; Uganda, E70, E75	Yes	Yes	[41,63,64]
B646L	p72, major capsid	Malta, Malawi, OURT88; NH/P68	Yes	NA	[41,63]
CP204L	p30, virus entry phosphoprotein	Malta, Malawi, OURT88; NH/P68	Yes	NA	[41,63]
E183L	p54, inner envelope	Malta, Malawi, OURT88; NH/P68; Uganda, E70, E75	Yes	Yes	[41,63,64]
K78R	p10, DNA- binding	Malta, Malawi, OURT88	NA	NA	[41,63]
Nonstructural proteins					
B602L	p72 chaperone	Malta, Malawi, OURT88; NH/P68; Uganda, E70, E75	Yes	Yes	[41,63,64]
F334L	Ribonucleotide reductase	Malta, Malawi, OURT88; NH/P68	Yes	NA	[41,63]
K196R	Thymidine kinase	Malta, Malawi, OURT88	NA	NA	[41,63]
NP419L	DNA ligase	Malta, Malawi, OURT88; NH/P68	Yes	NA	[41,63]
Unassigned proteins					
K205R	Unknown	Malta, Malawi, OURT88; NH/P68; Uganda, E70, E75	Yes	Yes	[41,63,64]
E184L	Unknown	Malta, Malawi, OURT88	NA	NA	[63]
CP312R	Unknown	Malta, Malawi, OURT88; NH/P68	Yes	NA	[41,63]
C44L	Unknown	Malta, Malawi, OURT88	NA	NA	[41,63]

NA = not available.

**Table 6:** Identified ASFV immunogens.

ASFV Gene	Product	Delivery Method	Antibody Response	T Cell Response	Reference
Structural proteins					
B438L	p49, capsid formation	Vector	Yes	Yes	[57,61]
B646L	p72, major capsid	Protein, DNA, Vector	Yes	Yes	[48,49,55,56,59,60]
CP204L	p30, virus entry phosphoprotein	Protein, DNA, Vector	Yes	Yes	[46,47,49,51,52,55,56,59,60,61]
CP530R	pp62, core shell polyprotein	DNA, Vector	Yes	Yes	[56,61]
	p15	Protein, DNA	Yes	No	[59,60]
	p35	Protein, DNA	Yes	No	[59,60]
CP2475L	pp220, core shell polyprotein: p150, p37, p14, p34	DNA, Vector	NA	Yes	[61]
D117L	p17, inner envelope	DNA, Vector	Yes	low	[61]
E120R	p14.5	DNA, Vector	Yes	NA	[61]
E183L	p54, inner envelope	Protein, DNA, Vector	Yes	Yes	[46,47,49,51,52,55,56,59,60]
E199L	j18L, virion protein	DNA, Vector	NA	Yes	[61]
EP402R	CD2v, outer envelope	Protein, Vector	Yes	Yes	[44,49,59,61]
H108R	Inner envelope	DNA, Vector	Yes	NA	[61]
KP177R	p22, outer envelope	Protein	Yes	Yes	[48,61]
O61R	p12, envelope	Protein	Yes	Yes	[49,61]
Nonstructural proteins					
A151R	viral replication	Vector	Yes	Yes	[57]
B119L	9GL, virus assembly	Vector	Yes	Yes	[57]
B602L	p72 chaperone	DNA, Vector	Yes	Yes	[57,61]
EP153R	C-type lectin	DNA, Vector	Yes	Yes	[49,61]
F1055L	Helicase	DNA, Vector	NA	Yes	[61]
G1211R	DNA polymerase	DNA, Vector	NA	Yes	[61]
L10L	KP117R-related	DNA, Vector	Yes	NA	[61]
MGF360-11L	KP362L	DNA, Vector	NA	Yes	[61]
MGF505-4R	NA	DNA, Vector	NA	Yes	[61]
NP419L	DNA ligase	DNA, Vector	NA	Yes	[61]
NP1450L	RNA polymerase subunit 1	DNA, Vector	NA	Yes	[61]
Unassigned proteins					
K205R/A104R		Vector	Yes	Yes	[57]
EP364R		DNA, Vector	NA	Yes	[61]
F317L		DNA, Vector	NA	Yes	[61]

NA = not available.

**Table 7:** ASFV immunogens associated with immune-enhanced pathology.

ASFV Gene	Product	Clinical Associations	Antibody Response	T Cell Response	Ref.
Structural proteins					
B438L	p49, capsid formation	Higher clinical scores	Yes;	NA	[61]
B646L	p72, major capsid	Chronic lesions	Yes, high level	NA	[41]
		Enhanced infection in vitro and disease in vivo	Yes	No	[60]
		Higher clinical scores	Yes, low level	Low	[61]
CP204L	p30, virus entry phosphoprotein	Chronic lesions	Yes, high level	NA	[41]
		Hyperimmunization associated with fewer protected pigs	Not detectable	Yes	[51]
		Higher viremia in pigs; enhanced infection in vitro	Yes	Yes	[52]
		Enhanced infection in vitro and disease in vivo	Yes	No	[60]
		Higher clinical scores	Yes, high level	Yes	[61]
CP530R	pp62, core shell polyprotein	Higher clinical scores	NA	Yes	[61]
	p15 and p35	Enhanced infection in vitro and disease in vivo	Yes	Few	[60]
CP2475L	pp220, core shell polyprotein: p150, p37, p14, p34	Higher clinical scores	NA	Yes	[61]
D117L	p17, inner envelope	Enhanced infection in vitro and disease in vivo	Not detectable	No	[60]
		Higher clinical scores	Yes, high level	Low	[61]
E120R	p14.5	Higher clinical scores	Yes	NA	[61]
E183L	p54, inner envelope	Chronic lesions	Yes, high level	NA	[41]
		Hyperimmunization associated with fewer protected pigs	Not detectable	Yes	[51]
		Higher viremia in pigs; enhanced infection in vitro	Yes	Yes	[52]
		Enhanced infection in vitro and disease in vivo	Yes	No	[60]
		Higher clinical scores	Yes, low level	Yes	[61]
E199L	j18L, virion protein	Higher clinical scores	NA	Yes, high	[61]
EP402R	CD2v, outer envelope	Hyperimmunization associated with fewer protected pigs	Not detectable	Yes	[51]
		Enhanced infection in vitro and disease in vivo	NA	No	[60]
		Higher clinical scores	Yes	Low	[61]
H108R	Inner envelope	Higher clinical scores	Yes	NA	[61]
KP177R	p22, outer envelope	Higher clinical scores	Yes	Yes	[61]
O61R	p12, envelope	Higher clinical scores	Yes	NA	[61]
Nonstructural proteins					
B602L	p72 chaperone	Higher clinical scores	Yes	Yes	[61]
EP153R	C-type lectin	Higher clinical scores	Yes, high level	NA	[61]
F1055L	Helicase	Higher clinical scores	NA	Yes	[61]
G1211R	DNA polymerase	Higher clinical scores	NA	YES	[61]
K196R	Thymidine kinase	Chronic lesions	Yes, high level	NA	[41]
L10L	KP117R-related	Higher clinical scores	Yes, high level	NA	[61]
MGF360-11L	KP362L	Higher clinical scores	NA	Yes	[61]
MGF505-4R	NA	Higher clinical scores	NA	Yes	[61]
NP1450L	RNA polymerase subunit 1	Higher clinical scores	NA	Yes	[61]
NP419L	DNA ligase	Chronic lesions	Yes, high level	NA	[41]
		Higher clinical scores	NA	Yes	[61]
Unassigned proteins					
CP312R		Chronic lesions	Yes, high level	NA	[41]
K205R		Chronic lesions	Yes, high level	NA	[41]
EP364R		Higher clinical scores	NA	Yes	[61]
F317L		Higher clinical scores	NA	Yes	[61]

NA = not available.
